# The First Cadenza Challenge: Perceptual Evaluation of Machine Learning Systems to Improve Audio Quality of Popular Music for Those with Hearing Loss

**DOI:** 10.1177/23312165251408761

**Published:** 2026-01-30

**Authors:** Scott Bannister, Jennifer Firth, Gerardo Roa-Dabike, Rebecca Vos, William Whitmer, Alinka E. Greasley, Simone Graetzer, Bruno Fazenda, Trevor Cox, Jon Barker, Michael A. Akeroyd

**Affiliations:** 1 School of Music, 171134 University of Leeds, Leeds, UK; 2 Hearing Sciences, School of Medicine, 170718 University of Nottingham, Nottingham, UK; 3 School of Computer Science, 7315 University of Sheffield, Sheffield, UK; 4 Acoustics Research Centre, 7046 University of Salford, Salford, UK

**Keywords:** music, hearing loss, hearing aids, machine learning, signal processing, audio quality, source separation

## Abstract

Music is central to many people's lives, and hearing loss (HL) is often a barrier to musical engagement. Hearing aids (HAs) help, but their efficacy in improving speech does not consistently translate to music. This research evaluated systems submitted to the 1^st^ Cadenza Machine Learning Challenge, where entrants aimed to improve music audio quality for HA users through source separation and remixing. The HA users (*N* = 53, ranging from “mild” to “moderately severe” HL) assessed eight challenge systems (including one baseline using the HDemucs source separation algorithm, remixing to original mixes of music samples, and applying National Acoustic Laboratories Revised amplification) and rated 200 music samples processed for their HL. Participants rated samples on *basic audio quality, clarity, harshness, distortion, frequency balance*, and *liking*. Results suggest no entrant system surpassed the baseline for audio quality, although differences emerged in system efficacy across HL severities. *Clarity* and *distortion* ratings were most predictive of audio quality. Finally, some systems produced signals with higher objective loudness, spectral flux and clipping with increasing HL severity; these received lower audio quality ratings by listeners with moderately severe HL. Findings highlight how music enhancement requires varied solutions and tests across a range of HL severities. This challenge provided a first application of source separation to music listening with HL. However, state-of-the-art source separation algorithms limited the diversity of entrant solutions, resulting in no improvements over the baseline; to promote development of innovative processing strategies, future work should increase complexity of music listening scenarios to be addressed through source separation.

## Introduction

Engagement with music is a central dimension of many people's lives, encompassing music-making and performance (Lamont, 2012; North et al., 2000; Small, 1999), attending live concerts in-person or virtually (Brown & Knox, 2017; Pitts, 2016; Swarbrick & Vuoskoski, 2023), and listening to recorded music (Bonneville-Roussy et al., 2013; Krause et al., 2015). Music can have wide-ranging positive effects for people, including improved social and mental health (Groarke & Hogan, 2016; Perkins et al., 2020), fulfilment and well-being (Croom, 2015), and maintenance of self-identity (DeNora, 1999; Saarikallio, 2019). Hence, it is important to address difficulties that may limit musical engagement.

Hearing loss (HL) affects more than 1.5 billion people globally, with numbers expected to increase to 2.5 billion by 2050 (World Health Organization, 2021). Hearing loss has negative effects on music perception and is a barrier to musical engagement and enjoyment. Such effects include poor pitch perception, issues with identifying and separating individual instruments, and inaudibility of quieter passages in music (Greasley et al., 2020; Hake et al., 2024; Moore, 2016; Siedenburg et al., 2020).

Hearing aids (HAs) are crucial in addressing HL difficulties, enhancing sound quality for both speech and music. Modern HAs utilize signal processing strategies (e.g., selective frequency amplification, multiband compression, noise reduction) to improve speech intelligibility and quality. However, these strategies do not consistently translate to comparable improvements for music signals and can introduce distortion, excessive loudness, and loss or overamplification of bass and treble frequencies (Madsen & Moore, 2014; Marchand, 2019). These differences are likely due to key distinctions between the signal characteristics of speech and music, including larger dynamic and frequency ranges in music (see Chasin & Russo, 2004). Some HA manufacturers have introduced music program settings to enhance music listening experiences; however, their overall effectiveness remains mixed (Looi et al., 2019; Madsen & Moore, 2014; Vaisberg et al., 2019).

An alternative approach is to consider how reproduced audio signals can be remixed or rebalanced, to improve the experiences of those with HL. This research aimed to evaluate signal processing systems, developed in the 1^st^ Cadenza Machine Learning Challenge (CAD1). CAD1 was a public challenge, in which entrants were tasked with creating systems to demix stereo pop music excerpts into individual instrument tracks (e.g., vocals, drums, bass, and other), and remix these to improve the audio quality of music for HA users.

### Music Audio Quality and Signal Processing

A key consideration for improved music listening experiences is perceived audio quality. Audio quality perception is multidimensional, and distinct from “music” qualities (i.e., compositional characteristics such as melody, harmony, form, and rhythm). Holistic measures of *basic audio quality* (BAQ) capture the overall perceptual impression of a sound signal (Bech & Zacharov, 2006), but this is underpinned by various combinations of perceptual attributes (Berg & Rumsey, 2003; Le Bagousse et al., 2014; Letowski, 1989; Pedersen & Zacharov, 2015; Pike, 2019). Most research on perceptual attributes of audio quality does not focus on HL (though see Gabrielsson & Sjögren, 1979; Narendran & Humes, 2003). However, a recent sensory evaluation study involving HA users developed a listener-driven consensus on the important perceptual attributes of music audio quality (Bannister et al., 2024). In this, 12 HA users first listened to a selection of music samples and provided three single-word terms to describe their perceptions of audio quality for each; this generated a perceptual space of 373 unique terms. Participants then navigated this perceptual space across three focus groups, removing synonyms, antonyms and less relevant terms, before identifying relationships and groupings across remaining descriptors. These groups were agreed and finalized by participants as key listener-driven attributes of BAQ and were labeled *clarity, distortion, harshness, spaciousness, treble strength, middle strength,* and *bass strength*.

Various studies on signal processing have attempted to address these factors and improve music listening experiences for those with HL. Work on amplitude compression has demonstrated that for music, there is a preference for linear amplification (Arehart et al., 2011; Hansen, 2002; Kirchberger & Russo, 2016; Van Buuren et al., 1999), and wide dynamic range compression compared to compression limiting (Davies-Venn et al., 2007). Other studies show a relationship between improved listener ratings of audio quality and emphasis of lower frequencies (Arehart et al., 2011; Franks, 1982; Vaisberg et al., 2021). Uys et al. (2012) found that nonlinear frequency compression increased music enjoyment for HA users, and Parsa et al. (2013) noted that this approach may be especially useful for significant HL, as a way of balancing audibility of higher frequencies and overall audio quality. Crucially, the results reported in these studies were often variable across individuals, highlighting the heterogeneity of HL types, causes, and experiences that needs to be considered (Drever & Hugill, 2023).

### Music Rebalancing and Object-Based Audio

Rebalancing the music signal or reproduction is an alternative approach to improving listening experiences. Studies of people with cochlear implants (CIs) indicate that there are preferences for fewer instruments in a music mix (Kohlberg et al., 2015), amplification of lead vocals relative to other instruments (Buyens et al., 2014; Pons et al., 2016), and broader spatial distributions of instruments (Althoff et al., 2024).

There is limited work on HL and HA users, and it is unclear whether findings from CI research will translate to HA users given the distinct qualities of the technologies. But in a recent study on remixing and HL (involving bilateral HA users), Benjamin and Siedenburg (2023) manipulated key parameters of popular music signals, including lead-to-accompaniment level ratio (LAR), low-to-high frequency balance, and an equalization transformation that linearly shifts the power spectrum of the factory mix away from or toward from a smooth reference spectrum (averaged over a large number of vocal and instrumental tracks), increasing or decreasing spectral sparsity (i.e., accentuation of peaks and notches in frequency power spectrum), respectively. Hearing loss listeners preferred a higher LAR than normal hearing listeners; bilateral HA users preferred boosted higher frequencies and increased spectral sparsity through equalization transformation when listening without their HAs; and there were positive correlations between increased HL severity and increased LAR and equalization transformation.

Despite a paucity of studies on music remixing for those with HL, there is a clear rationale for exploring these approaches derived from research on *object-based audio*. Object-based audio is a contemporary development in broadcast technology, in which audio is stored as individual sound objects with corresponding metadata and then mixed at the point of reproduction (Woodcock et al., 2018). This affords flexibility compared to traditional fixed audio reproduction, as sound content can be rebalanced and optimized for different scenarios. One application of object-based audio is to allow audiences to personalize their listening experiences through rebalancing audio tracks (Bleidt et al., 2015). It has potential for those with HL (Ward & Shirley, 2019), to address dimensions of personalization such as speech intelligibility (Paulus et al., 2015), spatial separation (Arbogast et al., 2005), and audio cue redundancy (Shirley et al., 2017). Ward (2020; see also Ward et al., 2018) explored the use of object-based audio to enhance broadcast accessibility for those with HL, proposing the concept of “narrative importance” as a way of hierarchically organizing audio cues in terms of their importance for engagement. Similar approaches may be possible for music listening and HL.

### Source Separation and Machine Learning

Object-based audio can allow manual rebalancing and altering of the sound sources (e.g., musical instruments) to reflect an individual's preference. But access to individual audio tracks is rarely possible, and manual rebalancing may not be a straightforward task for users. There are promising possibilities for automating this process, evidenced in work on intelligent music mixing (De Man et al., 2019), and the potential of machine learning (ML) approaches (Mourgela, 2023). A substantial field of research focusses on music source separation, aiming to separate music mixes into audio stems (i.e., individual submixes or groupings of audio sources representing instruments or ensemble components) with minimal loss of quality (Cano et al., 2019). This then affords the potential to rebalance audio stems and combines these to produce a new mix. Automating this demix/remix process may have positive effects on technologies and devices used to improve music listening experiences for those with HL. Such an approach can capitalize on the contemporary application of ML techniques in signal processing. For example, ML methods could demix music signals and remix audio stems in accordance with the HL characteristics of an individual listener.

### Aims

This research aimed to perceptually evaluate music rebalancing algorithms submitted as part of the CAD1 ML challenge to improve experiences of audio quality for listeners with HL.

## Methodology

### Design

An online listening test was designed in which participants rated 200 music samples in terms of audio quality and liking. These samples were generated by eight ML systems, submitted as part of the CAD1 ML challenge.

One of these systems was a “baseline” pretrained source separation algorithm (see below), and another was a “do nothing” system (i.e., processed signals were equal to original signals, with no amplification). As a demix/remix challenge, systems were developed to demix pop and rock music into *vocal*, *drums*, *bass*, and *other* (VDBO) audio stems, and then remix these to improve audio quality for listeners with HL (using audiometric data), who were listening through headphones and without HAs. Systems were trained and evaluated objectively through the Hearing Aid Audio Quality Index (HAAQI; Kates & Arehart, 2016), a metric for HAs, developed based on listening tests with three music samples (but no pop or rock) using mono headphone presentation of audio (Arehart et al., 2011). Objective HAAQI evaluations are presented in a companion paper (Roa-Dabike et al., 2025). Each participant received personalized versions of the 200 music samples, processed for their hearing profiles. The study followed a balanced repeated-measures design, in which participants were asked to provide the same number of ratings across all music samples and ML systems.

This study received ethical approval from the University of Leeds research ethics committee (approval number: FAHC 21-125). All underlying research data (alongside processed music signals) are available in anonymized format via a Zenodo release - https://zenodo.org/records/13271525.

### Baseline and Entrant ML Systems

**Figure 1.**
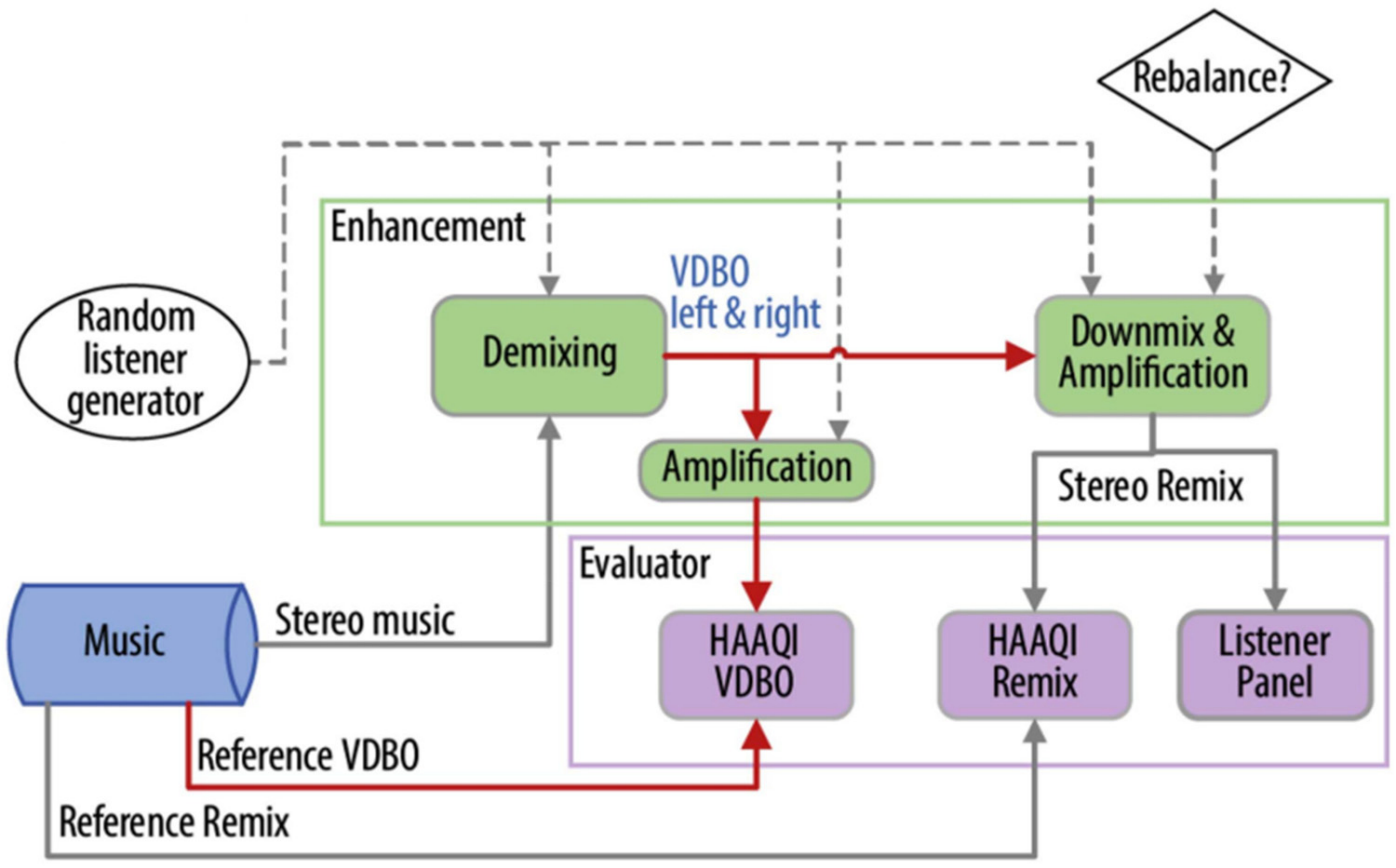
Architecture of the CAD1 baseline system (*Note:* VDBO = Vocals, Drums, Bass, and Other Audio Stem Representation).

The baseline system in CAD1 presented a solution to the demix/remix task outlined above, with entrants tasked with outperforming this system. The baseline architecture is visualized in Figure 1. This utilized the Hybrid Demucs (HDemucs) model (Défossez, 2021), which adopts a U-Net architecture to combine spectrogram-based and time-domain audio source separation. This is a commonly used out-of-the-box pretrained audio source separation algorithm and was used without retraining. For the frequency-dependent amplification stage to compensate for HL, the National Acoustic Laboratories Revised (NAL-R) linear HA prescription was used (Byrne et al., 2001), to match the default amplification applied to reference signals in HAAQI evaluations. Finally, the baseline system deployed a basic remixing strategy, which performed a linear addition of the amplified VDBO stems to create the remixed stereo output.

The baseline and entrant ML systems are briefly characterized in Table 1, demonstrating the source separation algorithms used, remix strategies deployed, and implementation of frequency-dependent amplification. Entrants were able to modify any strategy or process contained within the “Enhancement” box (green outline) in Figure 1.

**Table 1.** Overview of ML Systems and Approaches.

System	Separation	Remix	Amplification
E001*	HDemucs	Original	NAL-R
E005	Open-Unmix (Stöter et al., 2019) + Constant-Q Transform	Original	NAL-R
E012	HDemucs	Rebalanced (decrease nonvocal stems for moderate/moderately severe HL)	Multiband Compressor (attenuate “Other” audio stem)
E014	HDemucs	Original	NAL-R + decreased low-frequency attenuation
E016	Spleeter (Hennequin et al., 2020)	Original	NAL-R + Butterworth bandpass filter
E017	HDemucs	Midside EQ	NAL-R + Compressor
E021**	-	Original	None
E022	HDemucs	Rebalanced	NAL-R

* = baseline system; ** = “do nothing” system; HL = hearing loss; NAL-R = National Acoustic Laboratories Revised.

The “Separation” column names the source separation algorithm utilized. The “Remix” column specifies whether systems added audio stems together to create stereo remixes (i.e., “original”) or adopted other remixing approaches. The “Amplification” column outlines the approach taken to compensate for hearing loss, based on audiometric data.

System E005 used the Open-Unmix model (Stöter et al., 2019), which is a spectrogram-based audio source separation algorithm; this model was refined further through use of a sliced Constant-Q Transform (Holighaus et al., 2013), a neural network that uses a convolutional denoising autoencoder (Grais et al., 2021; Holighaus et al., 2013), and use of combined loss functions for training, such as CrossNet-Open-Unmix (Sawata et al., 2021).

E012 utilized a remixing strategy that decreased the level of nonvocal audio stems for moderate to severe HL. In addition, as the “other” audio stem can contain numerous different instruments, this system implemented a multiband compressor to attenuate this stem (in contrast to NAL-R amplification), with compression thresholds determined by levels of the “vocal” stem.

E014 largely resembled the baseline system, with use of HDemucs and the same remixing strategy. However, E014 used a modified NAL-R amplification, which decreased the attenuation of low frequencies in comparison to the original algorithm (i.e., 250 and 500 Hz bands were increased by 16 and 7 dB, respectively).

E016 used Spleeter (Hennequin et al., 2020), which contains a model pretrained on Deezer internal datasets for 4-stem music source separation, using U-Net architectures and spectrogram-based source separation. The system used NAL-R amplification and applied a Butterworth bandpass filter with −3 dB points at 240 Hz and 18.5 kHz.

E017 utilized the HDemucs algorithm but differed from the baseline system in terms of remix and amplification strategies. For remixing, this system implemented a mid-side equalization approach, which separates a stereo mix signal into central (i.e., mid) and stereo width (i.e., side) components. In separating the signal, filters were then incorporated to reduce the central signal by 2 dB below 2 kHz, and to increase the stereo width signals by 3 dB between 2 kHz and 6 kHz. For amplification, it used a single compressor for the remixed signal (threshold [RMS level above which compression begins, between 0 and 1] = 0.35; attenuation [mix factor between RMS and threshold for gain reduction] = 0.1, attack = 50 ms; release = 1000 ms; rms buffer size = 64 ms).

E021 was a “do nothing” system for comparison to entrants. This system did not perform any demixing or amplification and passed the original stereo music through unaltered.

Finally, E022 differed from the baseline system only in relation to remixing strategies. When all VDBO stems were concurrently nonsilent, this system applied gains of 7.6 dB to “vocals,” −8.0 dB to “drums,” and −4.4 dB to “bass” and “other” stems.

### Participants

The inclusion criteria were that participants were bilateral HA users, and between the ages of 18 and 90 years. Exclusion criteria included use of CIs or other hearing interventions besides HAs, diagnosis of Meniere's disease or hyperacusis, use of a programmable ventriculoperitoneal shunt, and severe tinnitus. These criteria were imposed to ensure safety and minimize discomfort through participation in the study.

**Figure 2.**
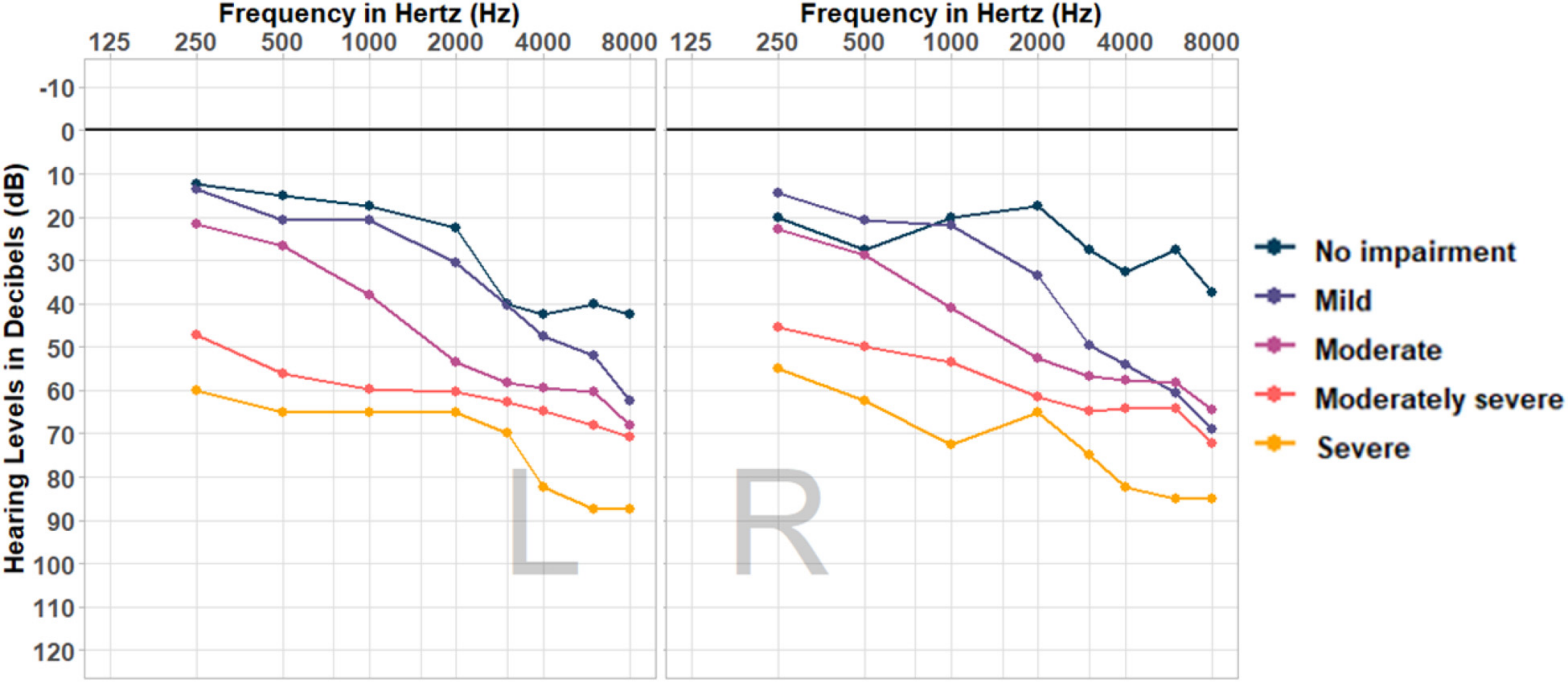
Average audiograms of participants, grouped by hearing loss (HL) severity. *Note:* HL severity labels correspond to categories derived from the World Health Organization's better ear four frequency average approach (Humes, 2019).

Fifty-three HA users completed the study (mean age = 67.0 years, SD = 15.0, 17 missing age responses; 20 females, 32 males, one missing sex response). Following the World Health Organization's four frequency average across 500 Hz, 1 kHz, 2 kHz, and 4 kHz for HL categorization (Humes, 2019), HL severities ranged across “no impairment” (*n* = 2; although participants were bilateral HA users with high-frequency loss), “mild” (*n* = 14), “moderate” (*n* = 18), “moderately severe” (*n* = 16), and “severe” (*n* = 3). Average thresholds for each HL severity are visualized in Figure 2. Audiograms were obtained by a trained researcher or provided by the participant as an audiogram measured by a professional audiologist within the 12 months prior to the listening test. Participants were recruited through an existing network interested in research on HAs and music, and through professional networks.

### Materials and Measures

#### Music Samples

Music samples were taken from the MUSDB18-HQ source separation dataset (Rafii et al., 2019). This contains 150 full duration music tracks (44.1 kHz sample rate) of varying popular genres, and their corresponding isolated VDBO audio stems. This VDBO representation is the standard format for music source separation. The dataset includes a defined evaluation set of 50 tracks that were used for the listening tests, although one track was excluded because it used offensive language. From the remaining 49 tracks, 25 were selected to limit the duration of the study and to ensure that participants could give ratings for all ML systems in a balanced repeated-measures design (25 samples × 8 systems = 200 samples to be rated). Tracks were selected by the research team to encapsulate a balance across genres represented in the dataset (pop/rock, electronic, heavy metal, rap, reggae), and a diversity in musical and audio characteristics. An excerpt of 15 s was randomly extracted from each full track. All four stems were active at some point within each excerpt. Entrant systems processed the 25 music samples separately for each participant's HL profile based on their audiometric data. Audiograms for seven participants were not available at the time of the challenge data release; for these participants, systems instead processed the samples based on the most similar audiogram available in the data release, determined by calculating the Euclidean distance between audiograms.

#### Audio Signal Features

Audio signal features were extracted from music samples using the Audio Toolbox functions (with default arguments unless specified below) in MATLAB (The Math Works, 2022). Spectrum features included centroid, entropy, flatness, flux, kurtosis, roll off, skew, slope, and spread; while other characteristics included crest factor (*peak2rms* function), RMS, and “objective loudness” based on ITU-R BS.1770-2 (we use the term “objective loudness” specifically to align with the terminology used by the International Telecommunication Union, in their standard for “objectively measuring the perceived loudness of audio signals”; it is not used to refer to subjective experiences of loudness in participants, but instead to emphasize a feature extracted from the signal). Additionally, a measure of “signal harshness” was extracted from audio signals, by taking the ratio of the power spectrum between 2 and 4 kHz and dividing this by the total power spectrum. Finally, a measure of signal clipping was extracted, by taking the inverse of the mean spectral kurtosis of the signal (see Prodeus et al., 2019). Features were selected to provide a theoretically driven and streamlined (i.e., limited number of features) set relevant to experiences of people with HL (Bannister et al., 2024; Greasley et al., 2020; Marchand, 2019). Features were extracted from the audio in 30 ms segments using a Hamming window to limit spectral leakage, with a 20 ms overlap. An average across these windows was calculated to represent the feature for the whole music sample.

#### Perceptual Measures

Participants rated each music sample for various aspects of audio quality. The aspects were derived from a study by Bannister et al. (2024), in which 12 HA users discussed and reached a consensus on key attributes of music audio quality across three focus group sessions. These listener-driven attributes were labeled *clarity, harshness, distortion, spaciousness, treble strength, middle strength, bass strength,* and *frequency balance*. In a pilot study of the current listening test, a subset of participants (*n* = 17) suggested that *spaciousness* and *middle strength* were difficult to rate. Given this, *spaciousness* and the three frequency attributes were dropped in the main listening study. Therefore, music samples were rated on 0–100 continuous scales for BAQ*, clarity, harshness, distortion,* and *frequency balance* (definitions in Supplementary File 1). Participants also reported their *liking* (0–100 scale) of the music samples, to accommodate possible differences between audio quality and preference ratings (Wilson & Fazenda, 2016) and emphasize this distinction for participants.

### Procedure

Potential participants were first prescreened through a short online questionnaire, to check inclusion and exclusion criteria. Eligible participants were sent an information sheet and provided informed consent to take part. Participants then attended a hearing test or provided researchers with an audiogram. Some participants engaged in an optional pilot of the listening study, to help refine task instructions, interfaces, and attribute ratings to be used. All participants were provided with a set of ADAPT 160 T USB II supra-aural headphones (EPOS, Denmark). While it was impossible to control the background levels at each participants’ location, using the same headphones offered some consistency to the testing.

Participants initially went through a series of checks to ensure that their headphones were working correctly and that HAs had been removed. Then, the perceptual attributes of audio quality were introduced, with definitions and rating scale structures; information was also given via a physical handout, for continued reference during the task (see Supplementary File 1). Next, participants listened to a few music examples (not from the 25 samples to be evaluated), to familiarize themselves with the task and set their volume levels so that the music was clearly audible but not uncomfortable. Participants then started the main task and rated 200 music samples in randomized order. Each sample was presented in stereo and could be heard as many times as required. The task took approximately 5 h to complete, and participants were encouraged to split the task into manageable blocks over a period of 6 weeks, to limit fatigue. Participants were reimbursed at an hourly rate.

### Data Analysis

The data were analyzed to investigate: (1) how BAQ scores differed between ML systems and HL severities; (2) the importance of perceptual attribute ratings for BAQ scores; (3) relationships between signal features of processed music samples and perceptual attribute ratings. This was performed using R (version 4.5.0; R Core Team, 2025). Correlations between BAQ ratings and HAAQI scores were also determined.

In the analysis of perceptual attribute and BAQ data, generalized linear mixed effects models were fitted with an ordered beta distribution family and logit link function. This method was used to accommodate bounded, continuous, zero-inflated distributions (Kubinec, 2023); these are distributions with a high total of zero scores, which was characteristic of the subjective rating data in this study (see Supplementary File 2 for a visualization of the BAQ data distribution). Models were fitted in R using the “glmmTMB” package (Brooks et al., 2017) and evaluated using the “performance” (Lüdecke et al., 2019) and “DHARMa” packages (Hartig, 2024). Post hoc pairwise comparisons were performed with Bonferroni correction, using the “emmeans” package (Lenth, 2024); with use of logit scaling, odds ratios were calculated in pairwise comparisons.

To investigate relationships between signal features of processed music samples and perceptual attributes, standardized signal feature data (*z-scores*, via “scale” function in R) were first subject to principal components analysis (PCA) using the “FactoMineR” package (Husson et al., 2024); parallel analysis and visualizations (e.g., scree plot) were utilized to determine the number of principal components (PCs) to retain. To confirm feature suitability for PCA, for any pair of signal features correlating at 0.90 or higher, only the feature with the highest sampling adequacy (via the Kaiser–Meyer–Olkin approach) was retained; subsequently, any feature with a sample adequacy lower than 0.60 was dropped from the analysis. This resulted in a set of eight signal features, summarized in Table 2. For every music sample for each participant, signal feature means were calculated and subsequently analyzed. Retained PCs were used as predictors of perceptual attribute ratings (*clarity*, *distortion*, *harshness*, and *frequency balance*) in generalized linear mixed effects models.

**Table 2.** Audio Signal Features Used in Principal Components Analysis.

Feature	Description	MATLAB function
*Spectral centroid*	Mean of the spectrum	*spectralCentroid*
*Spectral flux*	Variability of spectrum over time (between successive frames)	*spectralFlux*
*Spectral flatness*	Measure of noisiness vs. tonality in the spectrum (ratio of geometric mean of spectrum to arithmetic mean of spectrum)	*spectralFlatness*
*Spectral entropy*	Measures peakiness of the spectrum	*spectralEntropy*
*Spectral skew*	Skewness of spectrum distribution	*spectralSkew*
*Objective Loudness*	Objective measurement of the perceived loudness of audio signals	Custom function – computes according to ITU-R BS.1770-2
*Signal Harshness*	Ratio of spectral energy between 2 and 4 kHz to full spectrum	Custom function – power spectrum energy 2–4 kHz / total energy
*Clipping*	Inverse of spectral kurtosis in time domain (Prodeus et al., 2019)	1 / mean (*spectralKurtosis*)

To ensure that there was adequate sampling representation in each HL severity, “no impairment” and “mild” HL severities were combined into one group, and “moderately severe” and “severe” severities were combined. This resulted in three HL severities, namely “mild,” “moderate,” and “moderately severe.” All continuous perceptual ratings were rescaled from 0–100 to 0–1; for frequency balance ratings, which had an intuitive midpoint between bassy and trebly scale endpoints, this rescaling was achieved through computing a sine function for ratings multiplied by π divided by 100 (with rescaled values closer to 1 indicating balance between bass and treble).

## Results

### Descriptive Statistics

Table 3 provides descriptive statistics of BAQ and perceptual attribute ratings across the ML systems, and HL severity. Perceived audio quality decreases with increasing HL. The E014, E016, and E022 systems were rated with lower audio quality scores.

**Table 3.** Mean Scores (and Standard Deviations) of BAQ and Perceptual Attributes Across ML Systems and HL Severity.

	BAQ	Clarity	Harshness	Distortion	Frequency balance	Liking
ML System						
E001*	0.42 (0.25)	0.53 (0.27)	0.57 (0.28)	0.53 (0.29)	0.81 (0.24)	0.43 (0.23)
*E005*	0.42 (0.25)	0.53 (0.27)	0.57 (0.28)	0.53 (0.29)	0.81 (0.24)	0.43 (0.23)
*E012*	0.41 (0.26)	0.52 (0.27)	0.56 (0.29)	0.53 (0.29)	0.80 (0.25)	0.43 (0.23)
*E014*	0.33 (0.26)	0.41 (0.28)	0.56 (0.29)	0.63 (0.30)	0.77 (0.29)	0.40 (0.24)
*E016*	0.39 (0.23)	0.42 (0.25)	0.27 (0.22)	0.45 (0.28)	0.76 (0.27)	0.46 (0.21)
*E017*	0.42 (0.23)	0.52 (0.25)	0.35 (0.26)	0.43 (0.27)	0.85 (0.20)	0.47 (0.20)
E021**	0.43 (0.24)	0.46 (0.26)	0.25 (0.22)	0.41 (0.28)	0.77 (0.27)	0.49 (0.21)
*E022*	0.36 (0.24)	0.42 (0.27)	0.30 (0.25)	0.45 (0.28)	0.83 (0.22)	0.42 (0.21)
HL Severity						
*Mild*	0.45 (0.24)	0.53 (0.27)	0.39 (0.28)	0.45 (0.28)	0.85 (0.21)	0.46 (0.21)
*Moderate*	0.38 (0.24)	0.44 (0.25)	0.44 (0.29)	0.48 (0.29)	0.80 (0.26)	0.47 (0.19)
*Moderately Severe*	0.37 (0.25)	0.46 (0.28)	0.45 (0.31)	0.55 (0.29)	0.77 (0.27)	0.40 (0.25)

* = baseline system; ** = “do nothing” system; BAQ = basic audio quality; HL = hearing loss.

### Relationships Between BAQ Ratings and Objective HAAQI Scores

**Figure 3.**
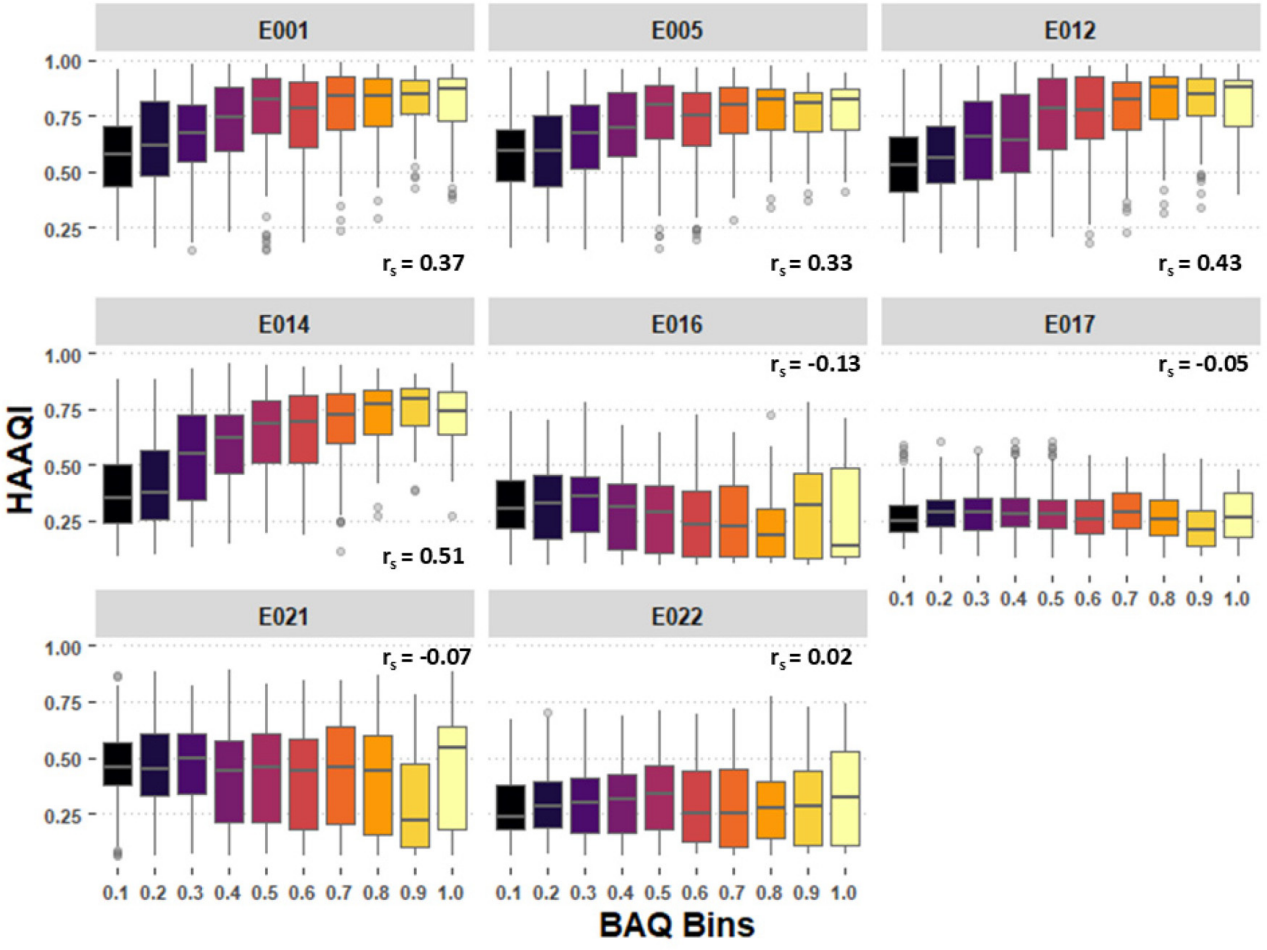
Boxplot visualization of relationships between basic audio quality (BAQ) ratings and Hearing Aid Audio Quality Index (HAAQI) scores for each machine learning (ML) system; *r_s_* values denote spearman correlations. *Note:* BAQ ratings (0–1) are collapsed into 10 bins for ease of visualization.

Figure 3 visualizes the relationships between BAQ and HAAQI for each ML system. Four systems showed a correlation between BAQ and HAAQI scores and four did not. This is related to the amplification used to compensate for raised hearing thresholds, and processes that rebalanced the VDBO stems before remixing. The HAAQI compares the processed signal to a reference (see Kates & Arehart, 2016), which in this case was the original stems added together and amplified by NAL-R, fed as input to a model of the impaired cochlea. If systems implemented other processes (i.e., in Table 1 where the remix was not “original” or the amplification was not “NAL-R”), then BAQ listener ratings might potentially increase, but HAAQI scores would decrease as the metric is based on the assumption that NAL-R amplification results in the highest possible audio quality. Most systems that did not have a correlation between BAQ and HAAQI did deviate from or modify the NAL-R amplification. Regardless, correlations remain modest for systems using NAL-R, which suggests an imperfect match of HAAQI to the current study data, and possible limitation of HAAQI in generalizing to the different music styles and stereo audio presentation used in this study.

### Basic Audio Quality Ratings Across ML System and HL Severity

**Figure 4.**
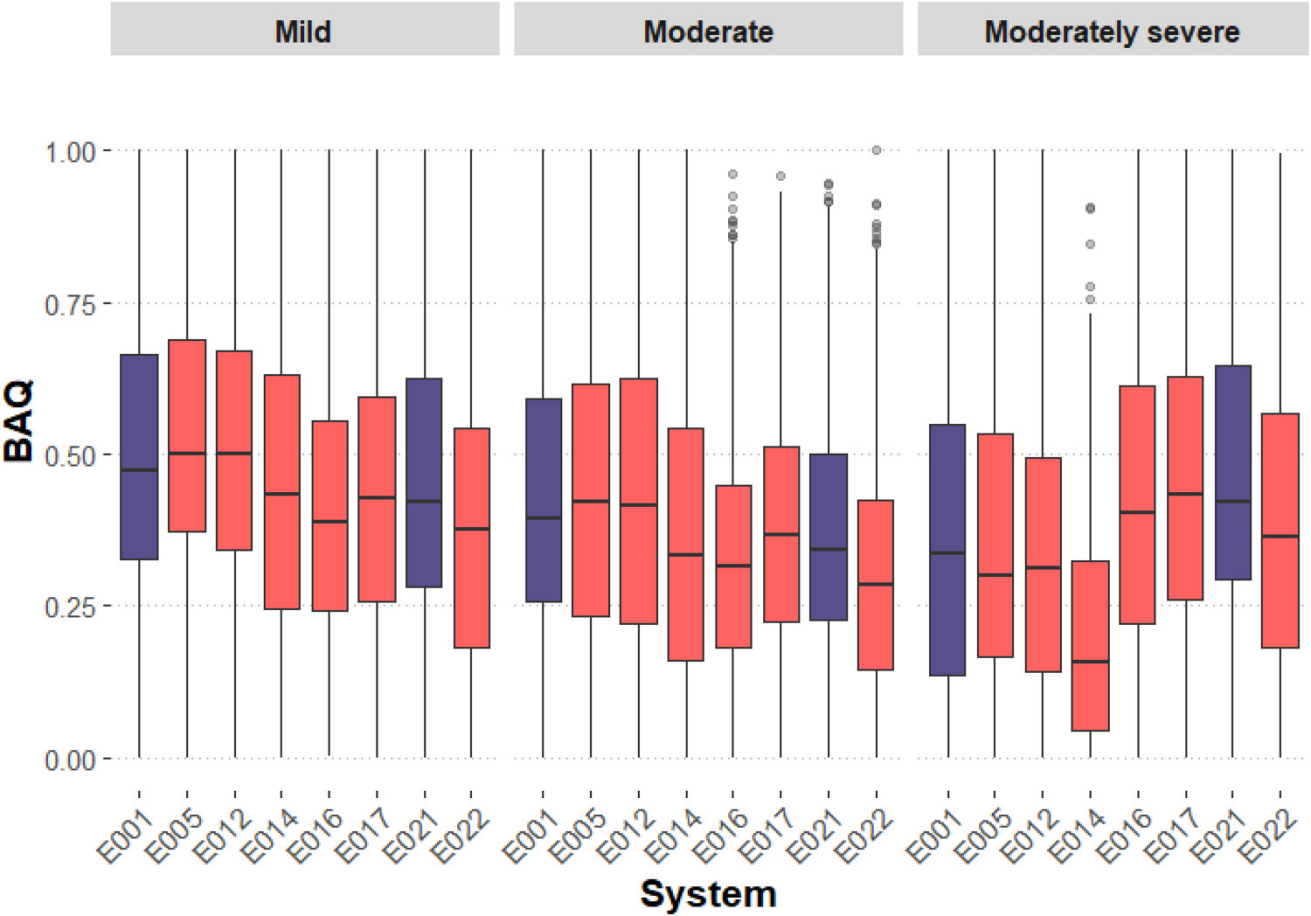
Boxplot of basic audio quality (BAQ) scores for each machine learning (ML) system, grouped by hearing loss (HL) severity. The purple boxes indicate “baseline” (E001) or “do nothing” (E021) systems.

To test differences in BAQ scores across ML systems and HL severity, a generalized linear mixed effects model was fitted. ML system and HL severity were fixed effects (including an interaction effect), and individual participant and music sample were fitted as random effects. Data are visualized in Figure 4, and the model is summarized in Table 4.

**Table 4.** Summary of the Generalized Linear Mixed Effects Model, with ML System and HL Severity as Fixed Effects.

Model summary	
AIC	−1223.00
BIC	−1012.60
ICC	.50
RMSE	0.20
*R*^2^ (marginal)	0.13
*R*^2^ (conditional)	0.60

Random effects		
	Variance	SD
Participant	0.12	0.34
Music sample	0.16	0.40

Fixed effects (intercept + significant effects)				
	β	SE	*z*	*p*
Intercept/Reference (System E001 / Mild HL)	−0.01	0.12	−0.14	.88
E014	−0.21	0.06	−3.39	<.001
E016	−0.33	0.06	−5.36	<.001
E017	−0.19	0.06	−3.22	.001
E022	−0.48	0.06	−7.67	<.001
Moderate HL	−0.28	0.13	−2.08	.036
Moderately severe HL	−0.58	0.13	−4.38	<.001
E014 * Moderately Severe HL	−0.48	0.08	−5.65	<.001
E016 * Moderately Severe HL	0.60	0.08	7.21	<.001
E017 * Moderately Severe HL	0.57	0.08	6.91	<.001
E021 * Moderately Severe HL	0.53	0.08	6.36	<.001
E022 * Moderately Severe HL	0.60	0.08	7.20	<.001

Only significant fixed and interaction effect coefficients are presented.

*Note:* AIC = Akaike Information Criterion; BIC = Bayesian Information Criterion; ICC = Intraclass Correlation Coefficient; RMSE = Root Mean Squared Error; HL = hearing loss.

Wald *x^2^* tests showed significant effects of ML system (*x^2^* = 257.43, *df* = 7, *p* < .001) and HL severity (*x^2^* = 9.47, *df* = 4, *p* < .001) on BAQ scores. Importantly, there was a significant interaction between ML system and HL severity (*x^2^* = 498.51, *df* = 14, *p* < .001). Post hoc pairwise comparisons were performed with Tukey adjustments to explore this interaction. Table 5 presents the results.

**Table 5.** Corrected Post Hoc Pairwise Comparisons Between HL Severities for Each ML System, in Terms of BAQ Scores.

System	Contrast: HL Severity	OR	95% CI	SE	z	p
E001*	Mild – Moderate	1.32	0.96, 1.82	0.18	2.08	.09
	**Moderate – Moderately Severe**	**1.34**	**1.00, 1.81**	**0.17**	**2.37**	**.** **04**
	**Mild – Moderately Severe**	**1.79**	**1.31, 2.44**	**0.23**	**2.37**	**.** **04**
*E005*	Mild – Moderate	1.37	0.99, 1.88	0.18	2.32	.05
	**Moderate – Moderately Severe**	**1.41**	**1.04, 1.89**	**0.17**	**2.72**	**.** **01**
	**Mild – Moderately Severe**	**1.93**	**1.41, 2.63**	**0.25**	**4.95**	**<.001**
*E012*	Mild – Moderate	1.32	0.96, 1.81	0.17	2.06	.09
	**Moderate – Moderately Severe**	**1.46**	**1.09, 1.97**	**0.18**	**3.03**	**.** **006**
	**Mild – Moderately Severe**	**1.93**	**1.41, 2.64**	**0.25**	**4.97**	**<.001**
*E014*	Mild – Moderate	1.29	0.94, 1.78	0.17	1.89	.14
	**Moderate – Moderately Severe**	**2.24**	**1.66, 3.03**	**0.28**	**6.34**	**<.001**
	**Mild – Moderately Severe**	**2.91**	**2.12, 3.98**	**0.39**	**7.94**	**<.001**
*E016*	Mild – Moderate	1.35	0.98, 1.85	0.18	2.21	.06
	**Moderate – Moderately Severe**	**0.72**	**0.54, 0.97**	**0.09**	**−2.54**	**.** **02**
	Mild – Moderately Severe	0.98	0.71, 1.33	0.13	−0.14	.98
*E017*	Mild – Moderate	1.31	0.95, 1.80	0.17	1.99	.11
	Moderate – Moderately Severe	0.76	0.57, 1.03	0.09	−2.09	.09
	Mild – Moderately Severe	1.00	0.73, 1.37	0.13	0.05	.99
E021**	**Mild – Moderate**	**1.51**	**1.09, 2.07**	**0.20**	**3.03**	**.** **006**
	**Moderate – Moderately Severe**	**0.69**	**0.51, 0.93**	**0.08**	**−2.86**	**.** **01**
	Mild – Moderately Severe	1.05	0.77, 1.43	0.13	0.38	.92
*E022*	Mild – Moderate	1.33	0.96, 1.83	0.18	2.09	.09
	**Moderate – Moderately Severe**	**0.73**	**0.54, 0.98**	**0.09**	**−2.47**	**.** **03**
	Mild – Moderately Severe	0.97	0.71, 1.33	0.12	−0.20	.97

* = baseline system; ** = “do nothing” system; BAQ = basic audio quality; HL = hearing loss.

Significant differences (*p* < .05) are in bold.

Results show that systems E001 (baseline), E005, E012, and E014 result in higher BAQ scores for mild or moderate HL levels than for moderately severe HL levels. However, there is a contrary trend for systems E016, E017, E021 (“do nothing”), and E022; these systems perform better for moderately severe HL levels than for moderate HL, although performance was similar between moderately severe and mild HL. A possible explanation for these results is that most systems losing performance with increased HL severity (E001, E005, E012) remix to the original mix *and* apply standard NAL-R amplification (see Table 1); this may result in changes to signal features with increasing HL severity that are not present for systems deviating from the E001 baseline in terms of remixing or amplification strategies (see “Music Signal Features and Perceptual Attributes” subsection below).

Post hoc pairwise comparisons were also performed between the eight systems, within each HL severity. Given the number of comparisons, the statistical output is provided in Supplementary File 3. However, interactions are shown in Figure 4. To summarize the system comparisons: (1) for mild and moderate HL severities, systems E001, E005, and E012 outperform the other five systems; (2) for moderately severe HL severity, systems E017 and E021 outperform systems E001, E005, and E012, with E014 performing poorly compared to all other systems.

### Basic Audio Quality, Perceptual Attributes, and Liking

To test relationships between BAQ scores, the perceptual attributes and liking of the music, a generalized linear mixed effects model was fitted. The four perceptual attributes (*clarity, harshness, distortion,* and *frequency balance*) and *liking* were fitted as fixed effects, and individual participant and music sample were fitted as random effects. The model summary is presented in Table 6.

**Table 6.** Summary of the Generalized Linear Mixed Effects Model, with Perceptual Attributes and Liking as Fixed Effects.

Model summary	
AIC	−10832.10
BIC	−10752.30
ICC	0.40
RMSE	0.13
*R*^2^ (marginal)	0.83
*R*^2^ (conditional)	0.90

Random effects		
	Variance	SD
Participant	0.07	0.27
Music Sample	0.004	0.06

Fixed effects				
	β	SE	*z*	*p*
(Intercept)	−1.63	0.05	−29.22	<.001
Clarity	2.16	0.03	68.52	<.001
Distortion	−1.18	0.03	−35.59	<.001
Frequency balance	0.60	0.03	19.52	<.001
Harshness	−0.27	0.03	−9.13	<.001
Liking	0.73	0.04	18.03	<.001

*Note:* AIC = Akaike Information Criterion; BIC = Bayesian Information Criterion; ICC = intraclass correlation coefficient; RMSE = root mean squared error.

**Figure 5.**
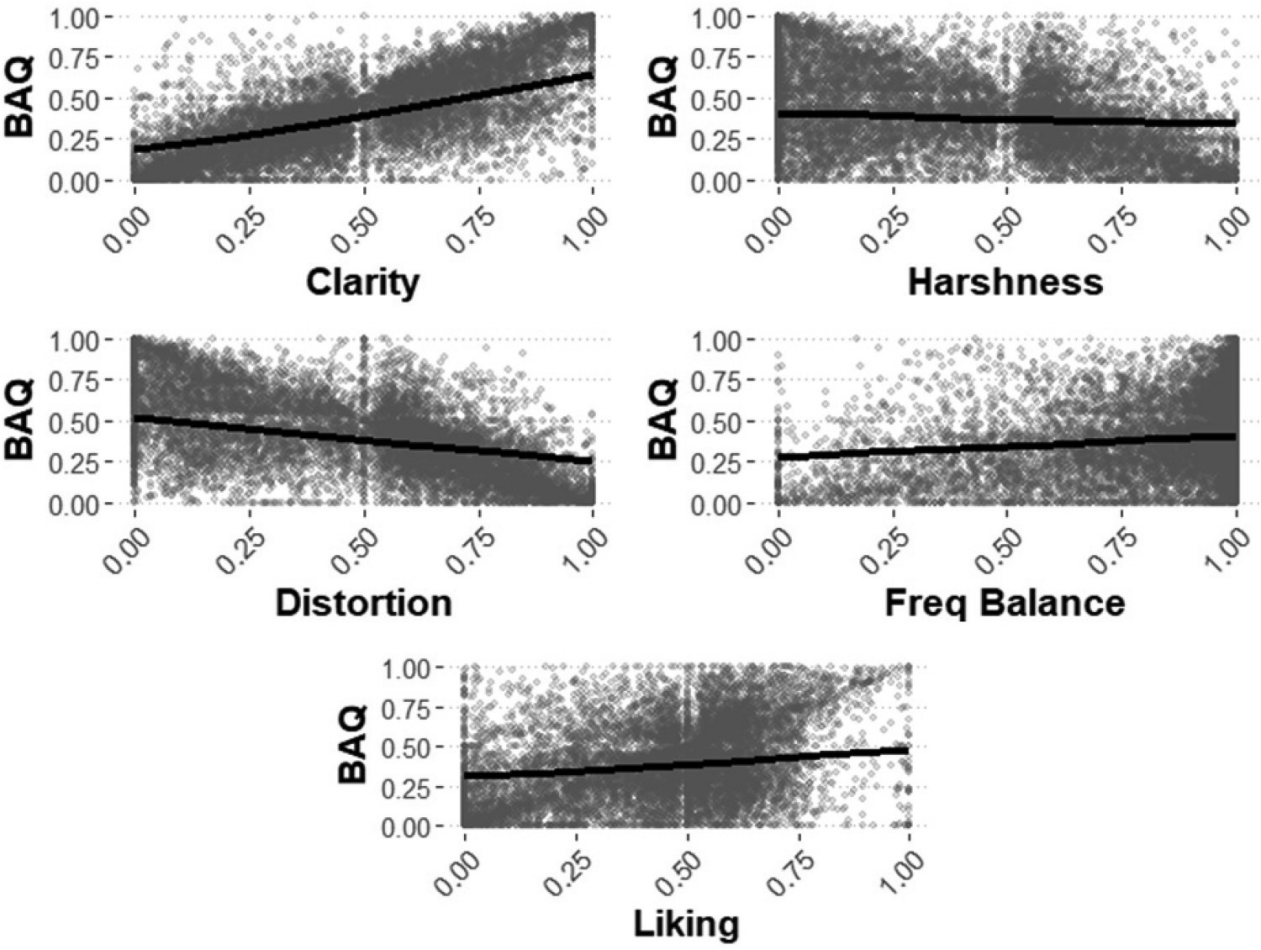
Scatterplot of raw basic audio quality (BAQ) scores, perceptual attributes and liking, with regression lines from the generalized linear mixed effects model.

Increases in *clarity* (β = 2.16, 95% CI [2.09, 2.22]), *frequency balance* (β = 0.60, 95% CI [0.54, 0.66]), and *liking* (β = 0.73, 95% CI [0.65, 0.82]) were associated with higher BAQ scores. Increases in *harshness* (β = −0.27, 95% CI [−0.33, −0.21]) and *distortion* (β = −1.18, 95% CI [−1.24, −1.11]) were associated with lower BAQ scores. The effects of *clarity* and *distortion* on BAQ scores were greater than the effects of *harshness*, *frequency balance*, and *liking*. Model outcomes are shown in Figure 5, alongside raw data.

### Music Signal Features and Perceptual Attributes

Eight audio signal features (see Table 2) were extracted from the music samples and regressed on to perceptual attribute ratings. Using Bartlett's test of sphericity, there was statistically significant data to reject the null hypothesis that the signal feature correlation matrix was equivalent to an identity matrix (*p* < .001), indicating that PCA could be performed. Similarly, the overall Kaiser–Meyer–Olkin measure of sampling adequacy was 0.76, above the recommended values of 0.60 (Hutcheson & Sofroniou, 1999). Finally, feature commonalities were all above 0.3, suggesting that each feature shared common variance with other features.

**Figure 6.**
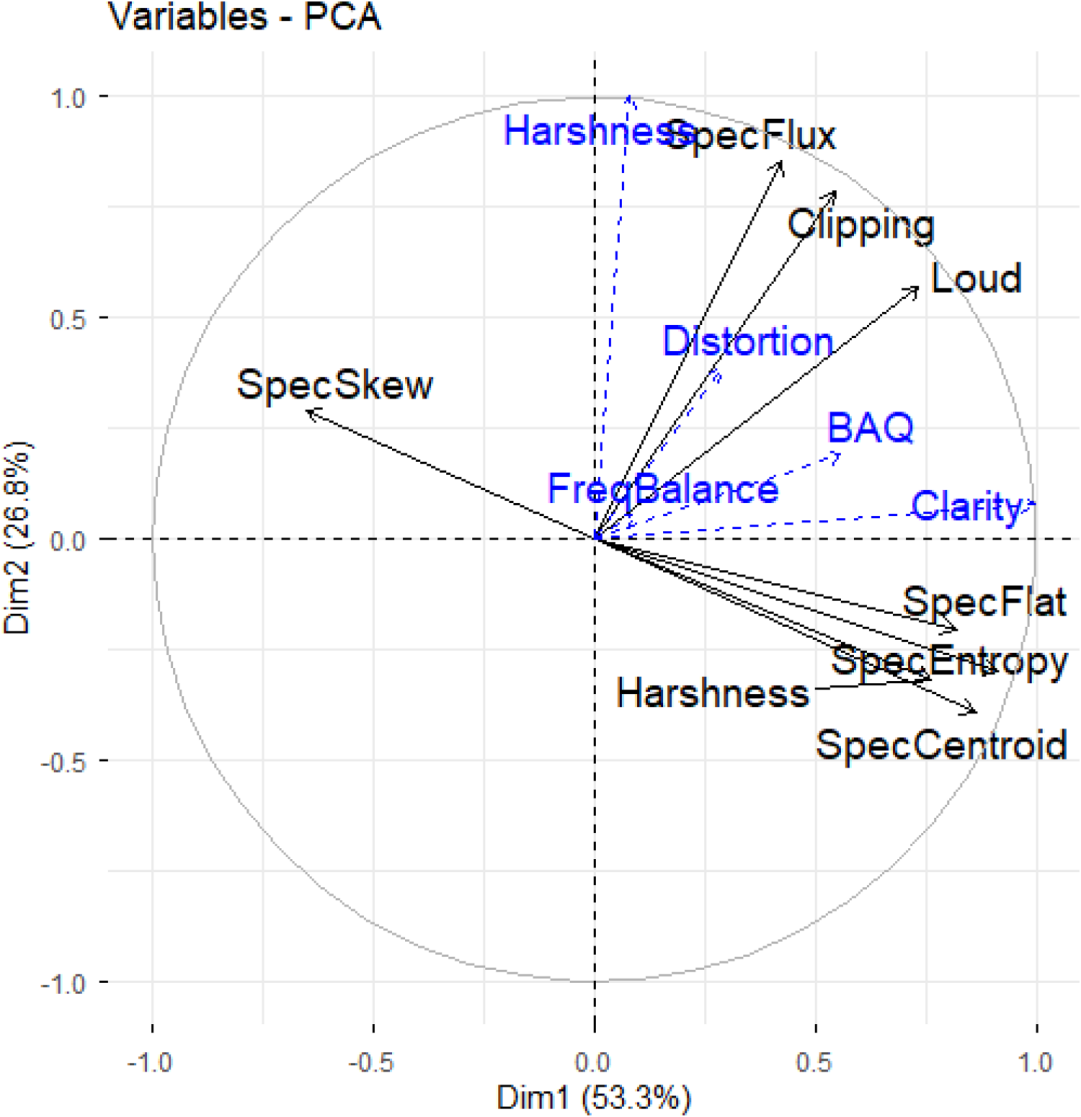
Plot of the first two principal components of the principal component analysis (PCA). *Note: SpecFlux* = Spectral Flux; *SpecSkew* = Spectral Skew; *SpecFlat* = Spectral Flatness; *SpecEntropy* = Spectral Entropy; *SpecCentroid* = Spectral Centroid; *FreqBalance *= Frequency Balance. Perceptual attributes of audio quality are included as supplementary variables, in blue font.

After checking eigenvalues, a scree plot and running parallel analysis, two components were retained, explaining 80.0% of the variance; these components are visualized in Figure 6.

A follow up PCA performed with a two-component solution and varimax rotation produced similar results (explaining 80% of the variance), with good fit (.96). Component loadings are displayed in Table 7. The first rotated component (RC1) was comprised of long-term spectral features of the audio signal (spectral entropy, spectral centroid, spectral flatness, and signal harshness); the remaining features (clipping, objective loudness, and spectral flux) loaded on to the second component (RC2) and reflect amplitude aspects of the signal, such as nonlinear distortions.

**Table 7.** Rotated Principal Component Feature Loadings.

Feature	RC1 (47.4%)	RC2 (32.7%)
Spectral entropy	**0.94**	
Spectral centroid	**0.94**	
Signal harshness	**0.82**	
Spectral flatness	**0.81**	
Spectral skew	−0.71	
Spectral flux		**0.95**
Clipping		**0.95**
Objective loudness	0.37	**0.84**

Values ≥ 0.80 (+/-) are in bold font. Percentages indicate proportion of variance explained for each RC.

**Figure 7.**
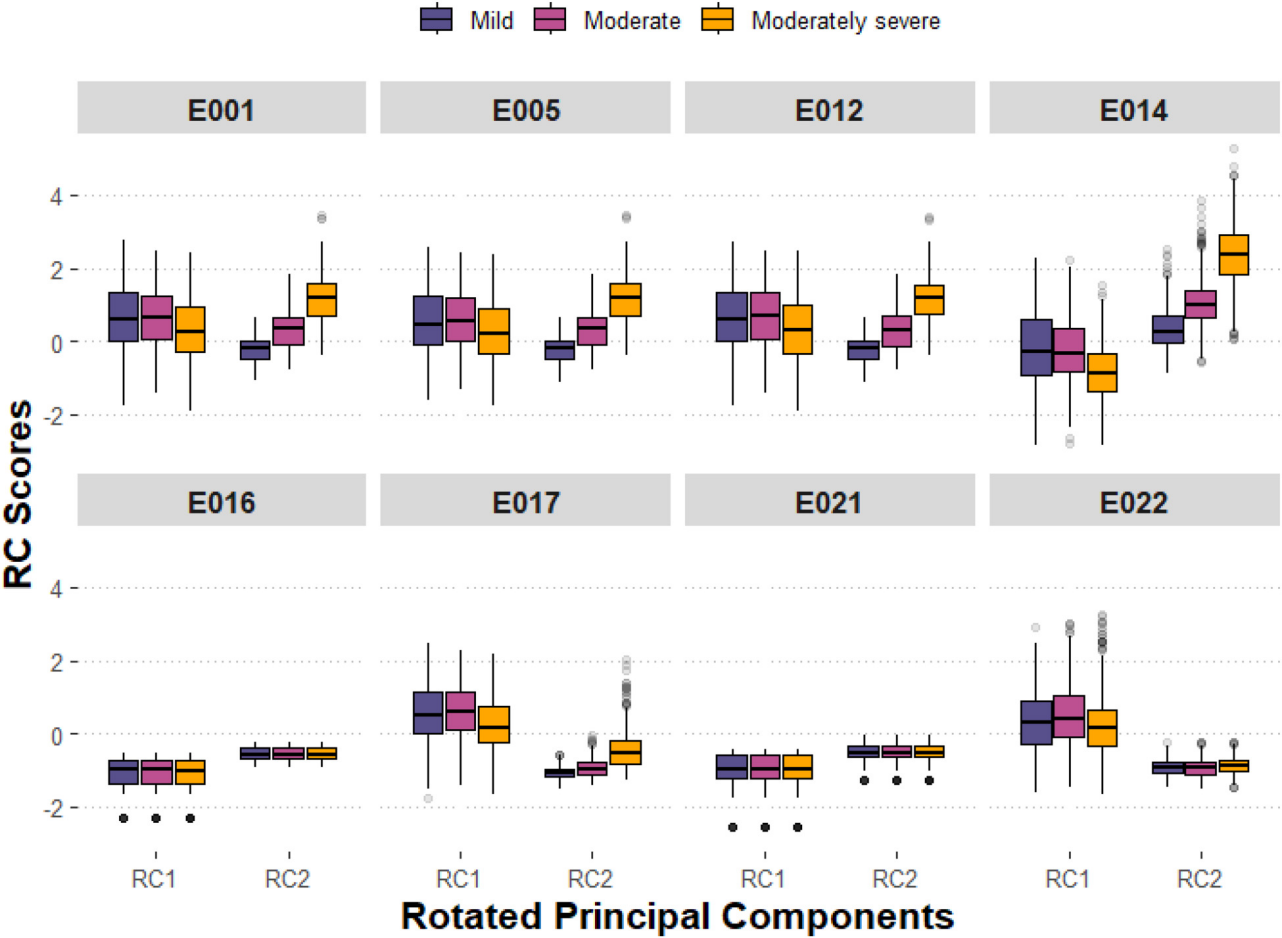
Boxplot of rotated principal component scores for each machine learning (ML) systems and each hearing loss (HL) severity.

Five generalized linear mixed effects models were fitted with RC1 and RC2 as fixed effects, and participant and song as random effects, to investigate relationships between signal features and BAQ and perceptual attributes (clarity, distortion, harshness, and frequency balance; see Supplementary File 1 for definitions). Model outputs are presented in Table 8. Results suggest that signal features may be more closely associated with ratings of distortion and harshness than with other ratings, driven largely by RC2 scores (i.e., higher objective loudness, clipping, and spectral flux). Although signal features were somewhat less related to clarity and frequency balance ratings overall, clarity was linked to higher RC1 scores (spectral centroid, spectral entropy, spectral flatness, and signal harshness), and frequency balance was related to lower RC2 scores. Finally, increases in BAQ were associated with higher RC1 scores and lower RC2 scores. Figure 7 provides a boxplot of rotated PC scores across systems, for each HL severity.

**Table 8.** Results of Generalized Linear Mixed Effects Models, with the Two RCs as Fixed Effects.

	Clarity	Distortion	Harshness	Frequency balance	BAQ
AIC	−32.30	1002.30	−820.50	−13648.20	−908.40
BIC	25.60	1060.20	−762.60	−13590.20	−850.50
ICC	0.60	0.70	0.60	0.60	0.50
RMSE	0.23	0.22	0.20	0.25	0.21
*R* ^2^_m_	0.09	0.24	0.47	0.06	0.07
*R* ^2^_c_	0.65	0.74	0.81	0.64	0.57
β^0^ (intercept)	−0.09	−0.03	−0.29**	1.00***	−0.40***
β^1^ (RC1)	0.19***	0.02	0.35***	0.01	0.06***
β^2^ (RC2)	−0.13***	0.43***	0.65***	−0.16***	−0.20***

*Note:* **p < .01; ***p < .001. AIC = Akaike Information Criterion; BAQ = basic audio quality; BIC = Bayesian Information Criterion; ICC = intraclass correlation coefficient; RMSE = root mean squared error.

Bold values reflect the largest fixed effect coefficients within each model.

**Figure 8.**
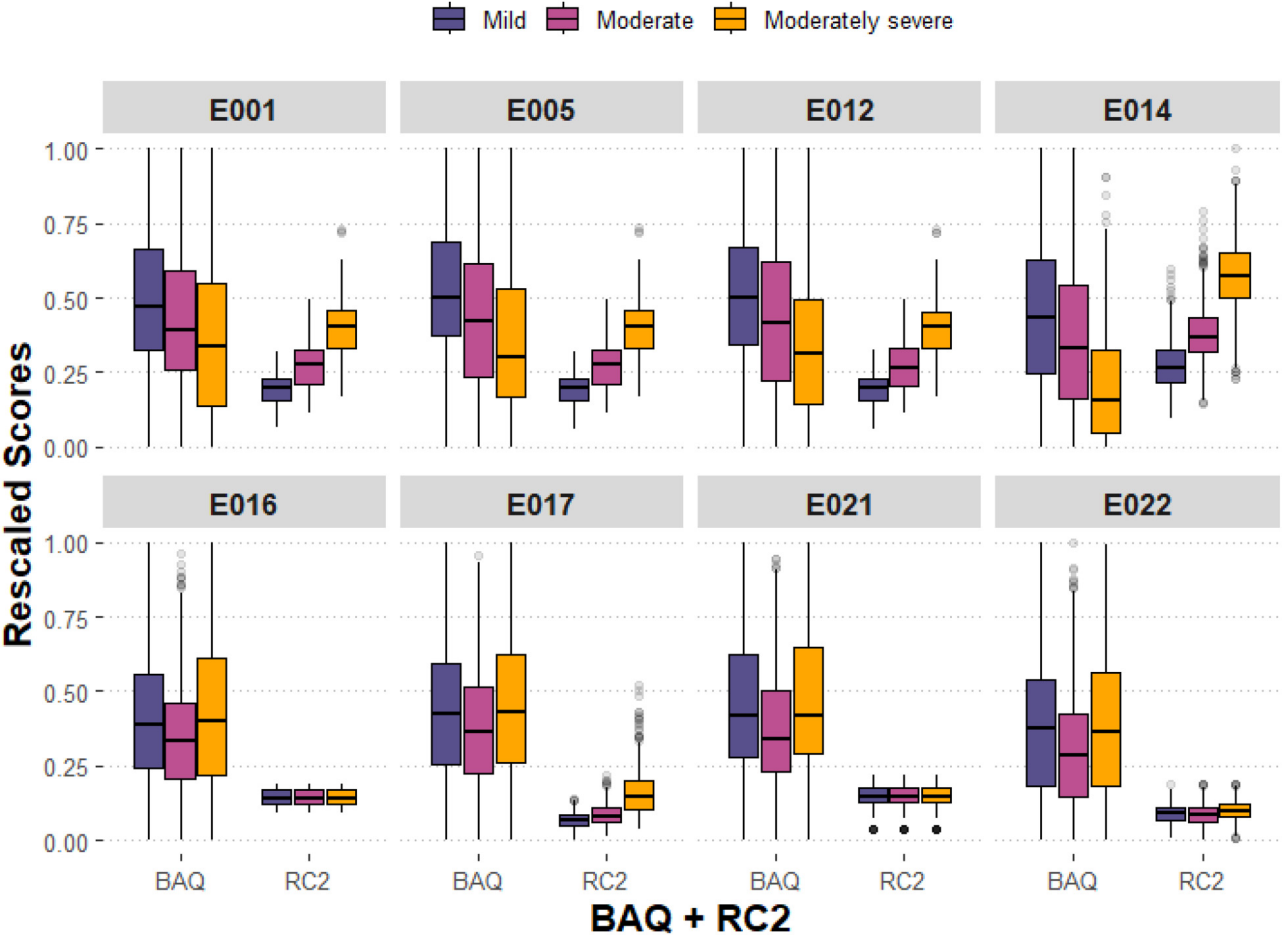
Boxplot of basic audio quality (BAQ) and RC2 scores for each machine learning (ML) system and each hearing loss (HL) severity. *Note:* RC2 data were rescaled to a range of zero and one, for the purpose of visual interpretation.

Systems E001 (baseline), E005, E012, and E014 demonstrate a pattern of increased RC2 scores (objective loudness, clipping, and spectral flux), with increasing HL severity. Given earlier analysis indicating that these four systems also perform more poorly with increased HL severity, Figure 8 shows BAQ data alongside RC2 scores for each system and each HL severity. With increasing HL severity, signal properties encapsulated by RC2 are increased and BAQ ratings decreased for E001, E005, E012, and E014; for the other four systems, there was little change in RC2 scores and BAQ ratings across the different HL categories.

## Discussion

The perceptual attributes of *clarity* and *distortion* were significant predictors of audio quality (BAQ); *harshness*, *frequency balance*, and *liking* also predicted BAQ but less strongly. In addition, signal features reflecting amplitude envelope distortion (clipping) were related to perceptions of *distortion* and *harshness*, whereas *clarity* was related to the homogeneity of the long-term spectrum of the audio signal. The ML systems varied significantly in rated performance, with the best rated challenge entrant systems not surpassing the E001 baseline or E021 “do nothing” systems in terms of BAQ. The BAQ scores varied significantly with HL severity: Some systems performed better for less severe HL, while other systems performed better for more severe HL. The following discussion interprets the results, outlines next steps and future directions in the research, and summarizes limitations of the study.

### Basic Audio Quality, ML Systems, and HL Severity

Systems E001 (baseline), E005, E012, and E021 (“do nothing”) received the highest BAQ ratings. Linear mixed effects modeling (Table 4) highlighted no significant differences between the E001 baseline performance (at mild HL severity), and E005, E012, or E021. It was found that systems E014, E016, E017, and E022 were significantly outperformed by the E001 baseline, although such direct comparisons are difficult in the presence of interactions with HL severity (see below). One conclusion from this 1^st^ Cadenza Machine Learning Challenge (CAD1) was that no entrant system surpassed the baseline solution. Notably, similar results were found for objective HAAQI evaluation (Roa-Dabike et al., 2025), with E001 achieving the highest mean HAAQI score for the remixed music signals. A possible explanation is that the baseline system utilized a state-of-the-art source separation model, HDemucs (Défossez, 2021), which upon reflection was difficult to outperform in a task where demixing processes are central. This focus on music source separation, with its highly developed algorithms, may also have limited the potential diversity of entrant solutions, as deviations from existing algorithms were unlikely to improve performance. The subsequent Cadenza ICASSP 2024 Challenge addressed these aspects by adding additional complexities to the challenge task to give more scope for improving on the baseline and for developing different solutions (Roa-Dabike et al., 2024); this was done by focussing on stereo reproduction of music over loudspeakers as the listening scenario, introducing the need for frequency-dependent mixing of audio stem components across left-right channels. These CAD1 results also highlight the difficulty of having HAAQI as a metric in a ML task, because the need to define a reference for HAAQI acts as a constraint on the entrants’ approaches.

The two entrant systems that matched baseline performance (E005 and E012) took different approaches: E005 utilized a different source separation algorithm (Open-Unmix, Stöter et al., 2019) but replicated the same remixing and amplification approaches; E012 used HDemucs but adopted an alternative remixing strategy and used a multiband compressor in the amplification phase. The E021 “do nothing” system also matched the E001 baseline. To better understand how different source separation, remixing and amplification approaches may achieve similar performance in music audio quality for HA users, and why a “do nothing” system performed similarly to the challenge benchmark, it is critical to navigate how ML system performance interacts with HL severities of the listener panel participants, as this underlies and elucidates overall performance.

Regarding BAQ ratings, an interaction effect was found between ML systems and HL severities, such that systems differ in terms of their performance across HL severities. In exploring these interactions, two broad groupings emerged across the eight systems (see Figure 4): E001, E005, and E012 outperformed other systems at mild HL severities but performed worse with increasing HL severity; contrastingly, E016, E017, E021, and E022 saw consistent performance for mild and moderately severe HL, and only reduced performance for moderate HL. E014 also performed best for mild HL but performance worsened with increasing HL severity with a notable drop in BAQ ratings for moderately severe HL. A similar grouping of systems emerges when evaluating audio signal features of the processed music samples across HL severities (see Figure 7). Although no pattern emerged in how the ML systems changed long-term spectral aspects of the audio (first PC) across HL severities, there were patterns when assessing amplitude envelope distortion (clipping) in the audio (second PC). For example, those systems performing best for mild HL but losing performance with increasing HL severity (E001, E005, E012, and E014) process music signals in a way that increases spectral flux, objective loudness and signal clipping, with increasing HL severity; in contrast, all other systems do not demonstrate this pattern yet maximize their performance for moderately severe HL. These differences are further inferred in Table 3, demonstrating that systems E001, E005, E012, and E014 were rated by participants as being perceptually harsher and more distorted, compared to the remaining systems. In relation to these ratings, one observation might be that across extended listening to music samples, participants may have acclimatized to linear processing but not to nonlinear distortions, although this remains conjecture.

These broader groupings can be interpreted by assessing the approaches taken by the ML systems (see Table 1). Regarding the first grouping (E001, E005, E012, E014), systems E001, E005, and E014 all use the NAL-R HA prescription for amplification, with E014 modifying this to reduce low-frequency attenuation. Studies have shown that preferred gains of individual HA users can vary above and below the values derived from fitting methods (Keidser et al., 2008; Keidser et al., 2012; Vaisberg et al., 2021), and NAL-R does not account for nonlinear dependencies in HL, like loudness recruitment. As such, use of this amplification strategy may relate to decreases in BAQ ratings with more severe HL. System E012 used HDemucs and a remixing strategy to decrease the levels of nonvocal stems (i.e., drums, bass, and other) for moderate to severe HL; for amplification, the system used a multiband compressor to attenuate the “other” stem, with compressor thresholds set based on the vocal levels. This approach also resulted in higher BAQ ratings for mild HL, decreasing with more severe HL, which may indicate shortcomings in the strategy of prioritizing vocals for HA users with moderately severe HL. Indeed, although hearing vocals and lyrics in music can be important for listeners (Barradas & Sakka, 2022), recent focus groups with HA users in the Cadenza Project highlight notable variability in the importance of these aspects: some individuals consider lyric intelligibility and hearing vocals to be central to their experience, whereas others may focus on different elements of the music. Critically, these aspects differ and are contingent on the styles of music being listened to (Condit-Schultz & Huron, 2015), with some styles (e.g., pop) positioning the vocal performance as central to the music. In the current context, it is possible that E012's prioritization of the vocal stems did not contribute to perceive audio quality improvements for the listener panel participants and introduced nonlinear distortions through compression.

Regarding the second grouping (E016, E017, E021, E022), E022 differed from the baseline in its remixing strategy, which increased gain of the vocal stems and decreased gain of the remaining stems (drums, bass, other), where all VDBO stems were concurrently nonsilent. This approach is similar in concept to E012 but uses NAL-R as opposed to multiband compression. However, the remixing strategy appeared to minimize increases in nonlinear distortions in music signals for more severe HL; although this afforded improved performance for moderately severe HL, the consistent gain strategy may have been detrimental for BAQ ratings for milder HL, possibly due to an overattenuation of the stems. E017 used HDemucs but adopted alternative remixing (mid-side EQ; attenuating a central signal component and increasing gain for stereo width signal component) and amplification strategies (single compressor), compared to the baseline system. This system showed only slight increases in distortions in signals for more severe HL; however, E017 was outperformed by most systems in the first grouping for mild HL, suggesting that the signal gains and compressor were not an improvement over NAL-R amplification. E016 used a different source separation algorithm (Spleeter; Hennequin et al., 2020), and a modified NAL-R amplification that applied a Butterworth bandpass filter (−3 dB points at 250 Hz and 18.5 kHz). This system did not process signals differently across HL severities, performed best for moderately severe HL, but was outperformed by the first grouping of systems for mild HL. Given the approaches taken by E016, it is plausible that the reduced performance compared to baseline may result from the alternative source separation algorithm used, especially given that the quality of the demixing was found to be lower in the objective HAAQI evaluation (Roa-Dabike et al., 2025).

It is important to consider the performance of the E021 “do nothing” system, which performed similarly to E001, E005, and E012. This finding suggests that the utility and potential of source separation and remixing approaches is currently unproven for listeners with HL (Benjamin & Siedenburg, 2023; Ward & Shirley, 2019). However, this may be a consequence of how the challenge was set-up, where a remixing of the signal back to the original stereo was the target for the objective evaluation by HAAQI. Furthermore, evaluating BAQ scores across systems does not capture the complexities and intricacies underlying the present data on audio quality, HL, and music signal processing, demonstrated by the statistical interactions between ML systems and HL severity. For instance, although no entrant system surpassed the E001 baseline solution overall, some systems *did* outperform the baseline for more severe HL. Research on music listening and HAs has demonstrated that severe HL exacerbates perceptual difficulties (Looi et al., 2019; Madsen & Moore, 2014); similarly, it is important to consider HL severity in understanding preferred processing strategies for music (Brennan et al., 2014). Ultimately, some entrant systems utilized strategies that maximized audio quality performance for moderately severe HL, which inevitably penalized overall performance as only 35% of the participant sample had moderately severe or severe HL.

These findings generate important insights relating to processing strategies and perceptual experiences of HA users with more severe HL. Results highlight that increasing sound levels with increasing HL severity (see Figures 7 and 8) may be ineffective for improving audio quality for these signals; this reflects existing literature suggesting that issues are not fully resolved through increased sound levels, due to issues of distortion and clipping, loudness recruitment, and feedback in some cases (Madsen & Moore, 2014; Moore, 2016). From a similar perspective, the overall performance of E021 (“do nothing”) raises the question of whether minimal or no processing of music can be effective, aligning with previous research reporting that some HA users prefer linear processing compared to nonlinear compression (Croghan et al., 2014; Kirchberger & Russo, 2016). Although one interpretation is that minimal signal processing is beneficial for music listening with more severe HL, another perspective is that alternative and personalized novel signal processing strategies are required for listeners with severe HL. To achieve that in ML, the training and evaluation datasets need to have greater representation from listeners with severe HL.

### Perceptual Attributes and Perceived Audio Quality

The current listening test generated extensive data about the listening experiences of people with HL, with the potential for informing the development of perceptual models of music audio quality. The definitions of BAQ and underlying perceptual attributes of audio quality were developed by a panel of HA users (Bannister et al., 2024), with definitions available in the Supplementary Material. The data highlight the importance of higher *clarity* and *liking*, and lower *distortion* for good audio quality for listeners with HL. *Harshness* was negatively related to BAQ, and *frequency balance* was positively related to BAQ. The relative importance of perceptual attributes for BAQ (Table 6) reflects the perspectives of the panel of HA users (Bannister et al., 2024), who considered *clarity* to be most important for music audio quality.

Importantly, perceptual attributes are linked to features of the music samples (Table 8). *Clarity* and BAQ are associated with higher spectral centroid, spectral entropy and spectral flatness. In contrast, *distortion* and *harshness* are linked to increases in level, clipping, and spectral flux, with *frequency balance* linked to decreases in these signal properties. These data and analyses provide an empirical foundation for predicting music audio quality in the context of HL, enabling future research into the development of a perceptual model suitable for ML approaches. This is important given the limitations with the current use of a double-ended objective measure such as HAAQI (as discussed above). A predictive model of audio quality based on audio signal features would provide a blind or single-ended measure, offering a route to ML optimizations that include HL, but is not constrained by the need to define a reference. Of course, such a model would be an empirical metric, with its applicability constrained within the bounds of the audio samples and listeners involved in this study. However, this is a key avenue for continued research, with the present perceptual data serving as a first step, to be extended through further perceptual data collected in a 2^nd^ Cadenza Challenge (CAD2).

### Future Directions

As key next steps in this program of research, further ML challenges have been carried out. The 2^nd^ Cadenza machine learning challenge (CAD2) involved two points of focus: (1) improvement of lyric intelligibility in music for listeners with HL; (2) rebalancing of classical music to improve audio quality. Lyric intelligibility may be a prevalent issue for listeners with HL (Greasley et al., 2020; Marchand, 2019), with notable effects for popular styles of music in which the vocals and lyrics are of central importance for musical enjoyment. As mentioned above, there are potential complex interactions between lyric intelligibility and perceived audio quality, as some listeners may place more importance on hearing lyrics compared to others. These differences may affect how signal processing strategies affect audio quality (such as those utilized by E012 in this study), and future work is needed to investigate this. It is also important to consider approaches and solutions for classical music listening, given that these styles may be important for older listeners (Bonneville-Roussy et al., 2013), a prominent demographic in HL communities given age-related patterns in HL.

### Limitations

There are limitations to the current listening test. Firstly, although participants were asked to listen to music samples at an audible and comfortable volume, it is not known what the playback levels were. The instructions aimed to reach a most comfortable level for each participant. However, it is likely that differences in playback volume remain, as numerous studies have demonstrated variability across individuals in preferred gains for HA fitting prescriptions such as NAL-R (Keidser et al., 2008; Keidser et al., 2012; Vaisberg et al., 2021); these differences may have had small effects on participant ratings beyond the ML systems and HL severities, but they also reflect more ecologically valid modes of listening, as listeners often have control over playback volume.

A second limitation is that not all participants listened to music processed specifically for their audiograms, but instead heard samples processed for the closest and most similar audiogram included in the CAD1 data release to entrants. However, participants who heard music samples processed for a similar audiogram demonstrated no unusual rating behaviors (e.g., markedly lower BAQ ratings), in relation to other participants. A further limitation is concerned with the music samples utilized in this study, which were derived from the MUSDB18-HQ dataset (Rafii et al., 2019). This enabled CAD1 to run using openly accessible datasets without restriction but imposed limits on the diversity of musical genres and styles used in the challenge. The resulting ML systems may not generalize to other types of music. Furthermore, feedback from participants indicated the need to include classical music. This limited scope of music genre and style reflects similar limitations across previous literature on music audio quality, HL, and signal processing (Arehart et al., 2011; Higgins et al., 2012). Future ML challenges and work on music and HL will need to consider diverse kinds of music as a priority. Finally, although this listening test involved a E001 baseline system and E021 “do nothing” system, interpretation of data would have been improved through the inclusion of a further baseline system which involved no source separation and no remixing, but included frequency-dependent amplification (i.e., NAL-R). This could have enabled a further disentangling of the separate effects of source separation, remixing, and amplification, to extend insights generated from the data. It is crucial that future challenges and associated listening tests consider this to help with analysis of the findings.

## Conclusions

The CAD1 provided a novel application of source separation technologies to the difficulties of music listening with HL. Listeners evaluated music signals that had been processed by different ML systems aimed at improving music audio quality for listeners with HL. Systems demixed and then remixed music samples, with listeners evaluating the processed music samples using perceptual attributes developed by a panel of HA users. The overall best performing ML systems matched the baseline system performance; additionally, most of these systems performed better for mild and moderate HL severity with poorer performance for moderately severe HL. These results, alongside the relative performance of the “do nothing” reference system, show that diverse and innovative approaches to ML and signal processing are needed (especially for severe HL), and that additional complexity in the processing task may afford more diverse entrant solutions, beyond existing state-of-the-art algorithms.

The listening test data demonstrate that perceptions of *clarity* and *distortion* are most important for BAQ, with *frequency balance* and *harshness* contributing to a lesser degree. Findings contribute to the continued development of alternative and novel signal processing strategies for music listening HL, and to our understanding of perceptions of audio quality by HA users.

## Supplemental Material

sj-docx-1-tia-10.1177_23312165251408761 - Supplemental material for The First Cadenza Challenge: Perceptual Evaluation of Machine Learning Systems to Improve Audio Quality of Popular Music for Those with Hearing Loss Supplemental material, sj-docx-1-tia-10.1177_23312165251408761 for The First Cadenza Challenge: Perceptual Evaluation of Machine Learning Systems to Improve Audio Quality of Popular Music for Those with Hearing Loss by Scott Bannister, Jennifer Firth, Gerardo Roa-Dabike, Rebecca Vos, William Whitmer, Alinka E. Greasley, Simone Graetzer, Bruno Fazenda, Trevor Cox, Jon Barker and Michael A. Akeroyd in Trends in Hearing

sj-docx-2-tia-10.1177_23312165251408761 - Supplemental material for The First Cadenza Challenge: Perceptual Evaluation of Machine Learning Systems to Improve Audio Quality of Popular Music for Those with Hearing Loss Supplemental material, sj-docx-2-tia-10.1177_23312165251408761 for The First Cadenza Challenge: Perceptual Evaluation of Machine Learning Systems to Improve Audio Quality of Popular Music for Those with Hearing Loss by Scott Bannister, Jennifer Firth, Gerardo Roa-Dabike, Rebecca Vos, William Whitmer, Alinka E. Greasley, Simone Graetzer, Bruno Fazenda, Trevor Cox, Jon Barker and Michael A. Akeroyd in Trends in Hearing

sj-tif-3-tia-10.1177_23312165251408761 - Supplemental material for The First Cadenza Challenge: Perceptual Evaluation of Machine Learning Systems to Improve Audio Quality of Popular Music for Those with Hearing Loss Supplemental material, sj-tif-3-tia-10.1177_23312165251408761 for The First Cadenza Challenge: Perceptual Evaluation of Machine Learning Systems to Improve Audio Quality of Popular Music for Those with Hearing Loss by Scott Bannister, Jennifer Firth, Gerardo Roa-Dabike, Rebecca Vos, William Whitmer, Alinka E. Greasley, Simone Graetzer, Bruno Fazenda, Trevor Cox, Jon Barker and Michael A. Akeroyd in Trends in Hearing
