# Supplementary material for: The First Cadenza Challenge: Perceptual Evaluation of Machine Learning Systems to Improve Audio Quality of Popular Music for Those with Hearing Loss
Source: Trends Hear. 2026 Jan 30;30:23312165251408761. doi: 10.1177/23312165251408761 (PMC12858752; doi:10.1177/23312165251408761)
Supplement: sj-docx-1-tia-10.1177_23312165251408761 - Supplemental material for The First Cadenza Challenge: Perceptual Evaluation of Machine Learning Systems to Improve Audio Quality of Popular Music for Those with Hearing Loss [file sj-docx-1-tia-10.1177_23312165251408761.docx]

# Audio Quality Assessment: Instructions

You will be asked to listen to some pieces of music and rate the audio quality of each in several ways as described below.

Overall Audio Quality (Very Poor to Very Good)

Perceived audio quality results from judgments of the sound of the music, in relation to a person’s expectations of how the music should ideally sound to them. Imagine listening to a piece of music in two different ways: listening through a cheap mobile phone, and then listening through high quality loudspeakers. The music is fundamentally same in both cases, but the audio quality is very different.

There are four audio quality attributes (see list below) and we would like you to use these attributes to describe the audio quality of the sample. We will also ask you to rate how much you liked the piece of music.

There will be five practice trials prior to starting the task.

You will notice that some of the music pieces will be repeated. However, the way the music is treated is slightly different each time, so please rate each as one "as is" without any comparison to the previous time you heard.

**Important:** You may also notice that the overall volumes of the pieces vary. In your ratings, please focus on the audio quality independent of the loudness.

**Please be aware** should you experience any difficulty (e.g., pain) with regards to loudness, please stop the experiment and notify the research team.

# Audio Quality Assessment: Definitions

Clarity (Very Unclear to Very Clear)

Clarity refers to how well you can hear the different elements of the music, including being able to distinguish between the different sound sources, instruments, or voices in the music, and being able to hear the qualities that distinguish one sound source from another. Unclear music may sound indistinct, mushy, or muddy; clear music may sound clean, distinct, and transparent.

Harshness (Not Harsh to Very Harsh)

Harshness refers to an emphasis or amplification of certain sound qualities (often in the treble frequencies or higher pitches) that can feel overwhelming, abrasive, painful, or discomforting. Harsh sounds may sound piercing, screechy, shrill, or sharp.

Distortion (Not Distorted to Very Distorted)

Distortion refers to a sense that the audio quality of the music contains elements that should not be there, that do not feel right, or that have appeared between the music’s reproduction and your listening of it.

These elements may include artefacts (e.g., noise, hiss, pops, crackles), or distortions to pitch (e.g., the pitches sound wrong compared to what you imagine was performed and recorded). No distortion may reflect a sense that the music is an authentic or accurate representation of what was performed and recorded, with no sense of pollution, interference, or distortion in the audio signal.

Frequency Balance (Very Bassy to Very Trebley)

Frequency balance refers to the perceived, relative balance between treble (or higher pitches of sound) and bass (or lower pitches of sound) in the audio. Audio described as more bassy would be characterised as having stronger or more prominent bass frequencies and pitches in comparison to treble frequencies and pitches, with the opposite true for audio described as more trebley. The middle point of this scale indicates a perceived balance between bass and treble.
