# Supplementary material for: The First Cadenza Challenge: Perceptual Evaluation of Machine Learning Systems to Improve Audio Quality of Popular Music for Those with Hearing Loss
Source: Trends Hear. 2026 Jan 30;30:23312165251408761. doi: 10.1177/23312165251408761 (PMC12858752; doi:10.1177/23312165251408761)
Supplement: sj-docx-2-tia-10.1177_23312165251408761 - Supplemental material for The First Cadenza Challenge: Perceptual Evaluation of Machine Learning Systems to Improve Audio Quality of Popular Music for Those with Hearing Loss [file sj-docx-2-tia-10.1177_23312165251408761.docx]

**Perceptual Evaluation of Machine Learning Systems to Improve Music Audio Quality for Hearing Loss**

**BAQ Ratings Across ML System and HL Severity – Post-Hoc Pairwise Comparisons between ML system for each HL severity level**

**Table S1 – ML system contrasts for mild HL levels (BAQ scores)**

| Contrast | *OR* | *SE* | *z* | *p* |
| --- | --- | --- | --- | --- |
| E001 – E014 | 1.24 | 0.07 | 3.58 | .008 |
| E001 – E016 | 1.41 | 0.08 | 5.67 | <.001 |
| E001 – E017 | 1.22 | 0.07 | 3.40 | .015 |
| E001 – E022 | 1.64 | 0.10 | 8.11 | <.001 |
| E005 – E014 | 1.32 | 0.08 | 4.62 | <.001 |
| E005 – E016 | 1.50 | 0.09 | 6.72 | <.001 |
| E005 – E017 | 1.30 | 0.07 | 4.45 | <.001 |
| E005 – E022 | 1.75 | 0.10 | 9.15 | <.001 |
| E012 – E014 | 1.25 | 0.07 | 3.77 | .003 |
| E012 – E016 | 1.42 | 0.08 | 5.87 | <.001 |
| E012 – E017 | 1.24 | 0.07 | 3.59 | .007 |
| E012 – E022 | 1.66 | 0.10 | 8.32 | <.001 |
| E014 – E022 | 1.32 | 0.08 | 4.52 | <.001 |
| E016 – E021 | 0.78 | 0.04 | -4.05 | .001 |
| E017 – E022 | 1.34 | 0.08 | 4.77 | <.001 |
| E021 – E022 | 1.49 | 0.09 | 6.52 | <.001 |

**Table S2 – ML system contrasts for moderate HL levels (BAQ scores)**

| Contrast | *OR* | *SE* | *z* | *p* |
| --- | --- | --- | --- | --- |
| E001 – E014 | 1.20 | 0.07 | 3.21 | .028 |
| E001 – E016 | 1.52 | 0.09 | 7.03 | <.001 |
| E001 – E017 | 1.32 | 0.07 | 4.68 | <.001 |
| E001 – E021 | 1.37 | 0.08 | 5.34 | <.001 |
| E001 – E022 | 1.84 | 0.11 | 10.09 | <.001 |
| E005 – E014 | 1.22 | 0.07 | 3.42 | .014 |
| E005 – E016 | 1.53 | 0.09 | 7.27 | <.001 |
| E005 – E017 | 1.33 | 0.07 | 4.90 | <.001 |
| E005 – E021 | 1.39 | 0.08 | 5.56 | <.001 |
| E005 – E022 | 1.86 | 0.11 | 10.34 | <.001 |
| E012 – E014 | 1.21 | 0.07 | 3.42 | .014 |
| E012 – E016 | 1.53 | 0.09 | 7.27 | <.001 |
| E012 – E017 | 1.33 | 0.07 | 4.89 | <.001 |
| E012 – E021 | 1.38 | 0.08 | 5.56 | <.001 |
| E012 – E022 | 1.85 | 0.11 | 10.35 | <.001 |
| E014 – E016 | 1.26 | 0.07 | 3.89 | .002 |
| E014 – E022 | 1.52 | 0.09 | 7.01 | <.001 |
| E016 – E022 | 1.21 | 0.07 | 3.12 | .038 |
| E017 – E022 | 1.39 | 0.08 | 5.43 | <.001 |
| E021 – E022 | 1.33 | 0.08 | 4.74 | <.001 |

**Table S3 – ML system contrasts for moderately severe HL levels (BAQ scores)**

| Contrast | *OR* | *SE* | *z* | *p* |
| --- | --- | --- | --- | --- |
| E001 – E014 | 2.05 | 0.12 | 11.49 | <.001 |
| E001 – E017 | 0.71 | 0.04 | -5.61 | <.001 |
| E001 – E021 | 0.76 | 0.04 | -4.38 | <.001 |
| E005 – E014 | 2.01 | 0.12 | 11.13 | <.001 |
| E005 – E017 | 0.70 | 0.04 | -5.91 | <.001 |
| E005 – E021 | 0.75 | 0.04 | -4.69 | <.001 |
| E012 – E014 | 1.87 | 0.11 | 9.98 | <.011 |
| E012 – E016 | 0.81 | 0.04 | -3.42 | .014 |
| E012 – E017 | 0.65 | 0.03 | -7.02 | <.001 |
| E012 – E021 | 0.70 | 0.04 | -5.80 | <.001 |
| E014 – E016 | 0.43 | 0.02 | -13.31 | <.001 |
| E014 – E017 | 0.34 | 0.02 | -16.79 | <.001 |
| E014 – E021 | 0.37 | 0.02 | -15.57 | <.001 |
| E014 – E022 | 0.51 | 0.03 | -10.30 | <.001 |
| E016 – E017 | 0.80 | 0.04 | -3.61 | .007 |
| E017 – E022 | 1.48 | 0.09 | 6.43 | <.001 |
| E021 – E022 | 1.38 | 0.08 | 5.23 | <.001 |
