# Supplementary figures and images for: The First Cadenza Challenge: Perceptual Evaluation of Machine Learning Systems to Improve Audio Quality of Popular Music for Those with Hearing Loss
Source: Trends Hear. 2026 Jan 30;30:23312165251408761. doi: 10.1177/23312165251408761 (PMC12858752; doi:10.1177/23312165251408761)

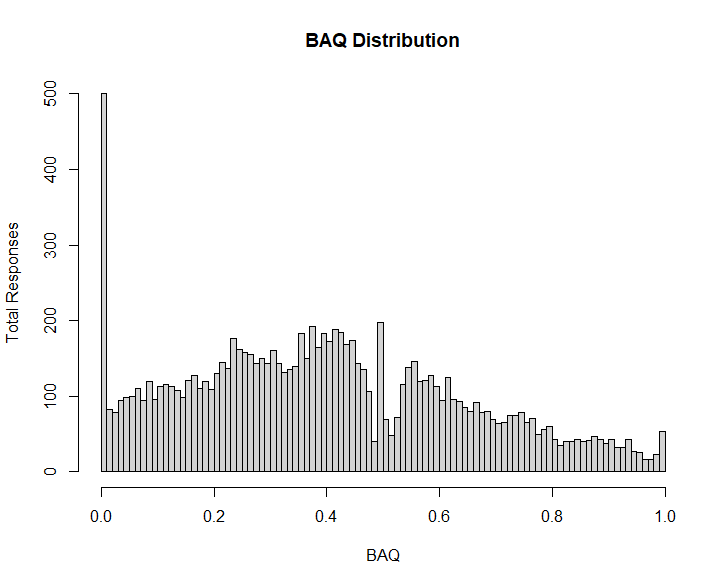

Supplement: sj-tif-3-tia-10.1177_23312165251408761 - Supplemental material for The First Cadenza Challenge: Perceptual Evaluation of Machine Learning Systems to Improve Audio Quality of Popular Music for Those with Hearing Loss [file sj-tif-3-tia-10.1177_23312165251408761.tif]
